# *Mamba*: a systematic software solution for beamline experiments at HEPS

**DOI:** 10.1107/S1600577522002697

**Published:** 2022-04-05

**Authors:** Yu Liu, Yan-Da Geng, Xiao-Xue Bi, Xiang Li, Ye Tao, Jian-She Cao, Yu-Hui Dong, Yi Zhang

**Affiliations:** a Institute of High Energy Physics, Chinese Academy of Sciences, Beijing 100049, People’s Republic of China; b Kuang Yaming Honors School, Nanjing University, Nanjing 210093, People’s Republic of China; c University of Chinese Academy of Sciences, Beijing 100049, People’s Republic of China

**Keywords:** *Bluesky*, experiment control, fly scans, high-throughput experiments, software architecture

## Abstract

To cater for the diverse experiment requirements at the High Energy Photon Source (HEPS) with often limited human resources, *Bluesky* was chosen as the basis for our software framework, *Mamba*. In our attempt to address *Bluesky*’s lack of integrated graphical user interfaces (GUIs), command injection with feedback was chosen as the main way for the GUIs to cooperate with the command line interface; a remote-procedure-call service is also provided, which covers functionalities unsuitable for command injection, as well as pushing of status updates. In order to fully support high-frequency applications like fly scans, *Bluesky*’s support for asynchronous control is being improved; furthermore, to support high-throughput experiments, *Mamba Data Worker* is being developed to cover the complexity in asynchronous online data processing for these experiments. To systematically simplify the specification of metadata, scan parameters and data-processing graphs for each type of experiment, an experiment parameter generator will be developed; experiment-specific modules to automate preparation steps will also be made. The integration of off-the-shelf code in *Mamba* for domain-specific needs is under investigation, and *Mamba GUI Studio* is being developed to simplify the implementation and integration of GUIs.

## Abstract 2

*Mamba* is a *Bluesky*-based experiment-control framework being developed for the High Energy Photon Source (HEPS); its frontend and backend collaborate through a remote-procedure-call service and, most importantly, command injection. Improvement of *Bluesky*’s support for high-frequency and high-throughput applications is in progress, with *Mamba Data Worker* as a key component. Other plans, including an experiment parameter generator and *Mamba GUI Studio*, have also been discussed.

## 1. Introduction

With the upgrade of synchrotron radiation facilities across the world, great progress is being continously made in providing X-ray beams with better emittance and coherence, as well as employing optical components and detectors with higher performance. Experiments with high communication frequencies or high data throughputs, as well as experiments involving multiple modes, complex *in situ* environments or automated changing of samples, are becoming increasingly prevalent. While allowing for multi-scale, multi-feature and *in situ* characterization of samples, this also poses fundamental challenges to experiment control and data acquisition/processing, both in experiments themselves and in the preparation steps before them. The large numbers of beamlines at many facilities, especially new facilities under construction, also result in the hard demand to implement diverse experiment requirements with a manageable codebase. At the High Energy Photon Source (HEPS) (Jiao *et al.*, 2018 ▸), a fourth-generation synchrotron radiation facility, where 14 beamlines will be provided in 2025 in its Phase I and up to 90 beamlines in total can be served in further phases, all issues above are to be expected. In order to address these issues, while keeping our codebase maintainable with often limited human resources, proper architecture design must be carried out for the software components involved.

The foundation of *Mamba*, our software framework, is the Python-based *Bluesky* (Allan *et al.*, 2019 ▸); before making the choice, we researched multiple well known alternatives for similar applications, like *GDA* (Gibbons *et al.*, 2011 ▸), *Sardana* (Coutinho *et al.*, 2011 ▸), *Karabo* (Hauf *et al.*, 2019 ▸) and *Py4Syn* (Slepicka *et al.*, 2015 ▸). Here we avoid discussing the details of our choice and instead note that the choice is not based on the availability of readily usable features but based on the total efforts needed to adapt the publicly available codebase to our applications. When saying ‘total efforts’, we not only include the efforts in development and maintenance of our own codebase but also include those in understanding, fixing and customizing the provided codebase. After our research, we concluded that, because of the quite well designed device interfaces (classes from the *ophyd* component of *Bluesky*) in conjunction with the simple yet relatively powerful mechanism to combine them in interlocked actions (RunEngine from *Bluesky*’s *bluesky* component) and represent extracted data in a friendly format (the ‘documents’) in real time, *Bluesky* is likely to fulfill a satisfactory fraction of the requirements at HEPS with the best cost-to-effect ratio. The first issue with *Bluesky* is the lack of integrated graphical user interfaces (GUIs); we discuss our approach to this issue in Section 2. For other challenges we note in the above, we give our plan of ongoing development in Section 3.

## 2. *Mamba*’s backend and frontend

**Figure 1:**
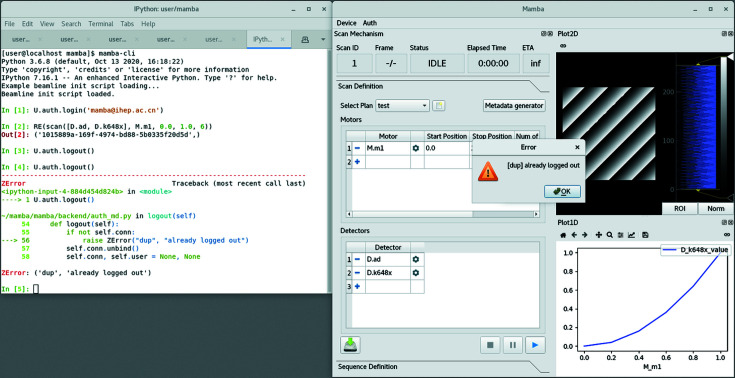
Command injection in *Mamba*, showing an error caused by attempting to log out twice after a successful experiment session; we explicitly note that the appearance of the GUIs may vary in the future due to ongoing frontend refactoring.

The first issue we observe with *Bluesky*, in comparison with its easily composable programming interfaces, is the lack of integrated GUIs. For some experiment tasks, this is more like just an obstacle to users with a relatively weak background in programming, but there are also tasks that are fundamentally easier with GUIs than only with keyboards. One good example is a requirement from the hard X-ray high-resolution spectroscopy beamline (B5) at HEPS [*cf.* also Huotari *et al.* (2017 ▸)], where many regions of interest (ROIs) need to be specified that properly cover individual light spots in sample images taken from area detectors (and light spots in images that will follow); another example is the manipulation of data-pipe graphs for *Mamba Data Worker* (*MDW*) (*cf.* Section 3). Since *Bluesky* recommends the IPython (Pérez & Granger, 2007 ▸) interactive command line interface (CLI) of Python for its regular use on beamlines, we designed *Mamba* with cooperation between the CLI (*Mamba* backend) and our GUIs (*Mamba* frontend) in mind; inspired by *AutoCAD*-like software (called ‘parametric modeling software’ in its industry), we extensively use what we call ‘command injection’ (Fig. 1 ▸) to implement this cooperation. Before discussing the communication architecture of *Mamba* in detail, we note that its frontend is still very immature in terms of internal structure and robustness; however, the architecture between the backend and frontend, which is the subject of this section, has been tested successfully in a real tomography experiment on a testbed for HEPS.

With command injection, we basically treat GUIs as ‘code generators’: most user operations with GUIs are translated into equivalent commands that get injected into the CLI, where they are actually executed. This design is beneficial in many ways, in terms of both user friendliness and architectural soundness. Users can naturally learn to use the CLI from using GUIs, and those more proficient in programming may abstract repeated tasks into succinct yet reusable CLI snippets. Sometimes, in order to perform some tasks that are not yet implemented or simply inflexible with GUIs, developers may even ask users to execute a few lines of code in the CLI; this is particularly meaningful when considering that developing the GUI for an application is typically much more complex than developing its CLI counterpart. The emphasis of GUIs as code generators also helps developers to naturally design optimal interfaces when implementing requirements, which increases modularity and consequently facilitates maintenance (especially automated testing). To summarize the above, command injection allows the CLI and GUIs to complement each other constructively, reducing workload for both users and developers.

**Figure 2:**
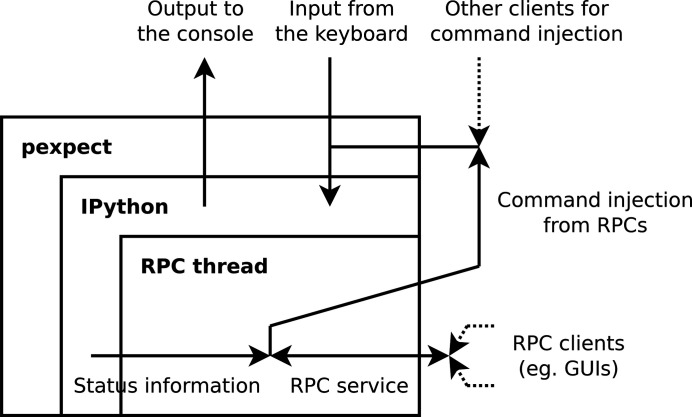
*Mamba*’s communication architecture.

**Figure 3:**
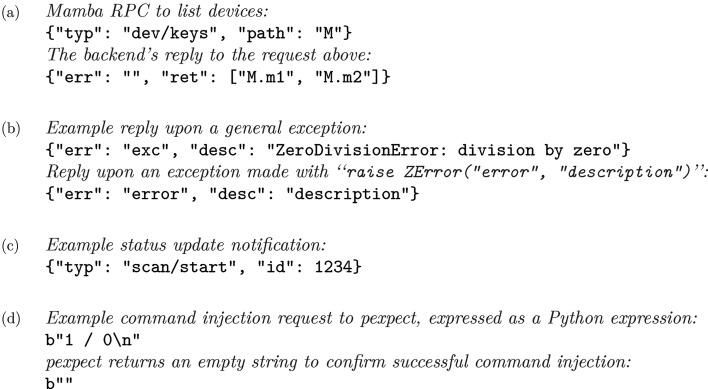
Example *Mamba* communication: (*a*) normal request/reply, (*b*) replies upon errors, (*c*) notification and (*d*) raw communication to *pexpect*; except for (*d*), which uses raw byte strings, all other (RPC) communication uses a JSON-based format.

**Figure 4:**
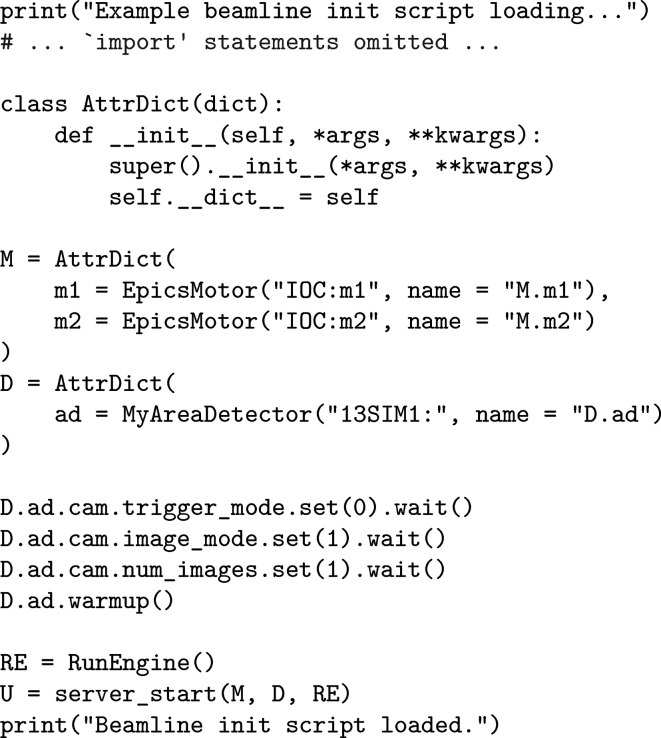
An example IPython startup script for *Mamba*.

The actual communication architecture between the backend and frontend of *Mamba* is shown in Fig. 2 ▸. The backend is run as a subprocess of a wrapping program, which is based on the *pexpect* library (https://pypi.org/project/pexpect) and forwards input from the user and output from the subprocess; the forwarding program also listens on a socket (*ZeroMQ* REP, https://zeromq.org/) that ‘command-injection clients’ can connect to, which sends the actual injection requests [Fig. 3 ▸(*d*)]. This way, commands are injected as if they were from the user’s keyboard. A problem with *pexpect*-based command injection is the lack of feedback: because the wrapping program does not understand the input semantics of the program (*e.g.* IPython) it wraps, it only knows whether some command has been successfully injected, instead of the final result (return value or exception in Python, *cf.* Fig. 1 ▸) of its execution. For this reason, we encapsulate command injection with a remote procedure call (RPC) service started by the server_start function in the *Mamba*-specific startup script for IPython (Fig. 4 ▸); the RPC service is a native Python thread with access to relevant IPython interfaces, so it can capture the results of injected commands and return them to its clients.

So the *Mamba* backend provides an RPC service that supports command injection with feedback; however, there are also communication requirements between the backend and frontend that are unsuitable for command injection. The first example is passwords, which should not appear in clear text on the CLI because of the command-line-history mechanism; additionally, many status queries (*e.g.* listing known motors and detectors) are only required by GUIs, and are inessential for CLI-only use of *Mamba*, so the appearance of relevant commands on the CLI would be mostly useless to users. Therefore we also provide ‘special RPCs’ for these requirements [Fig. 3 ▸(*a*)]; however, because RPCs need dedicated encapsulation code (RPC-specific syntax checks *etc.*), we have formed the policy that special RPCs should usually not be added for communication essential for CLI-only use. Noticing the need for the frontend to get status updates [Fig. 3 ▸(*c*)] and the intrinsic weakness of polling (polling too often wastes system resources and too infrequently risks loss of updates), in addition to a request/reply socket (*ZeroMQ* REP) for regular RPCs, a notification socket (*ZeroMQ* PUB) is also provided by the RPC service to proactively push updates to clients.

To further simplify our codebase, the finer details in *Mamba* have also undergone careful design, for which we give two examples. One is the introduction of ZError, a subclass of Python’s Exception, that can be raised by handlers in the RPC service to give fine-grained reports for errors that have been anticipated by developers [*cf.* Figs. 1 ▸ and 3 ▸(*b*)]. The RPC service will return information extracted from ZError to clients, instead of the more generic information it will return upon other types of exceptions; this way, RPC clients can set up exception handlers accordingly and only use a generic handler as a last resort. Another example is the use of variables M and D to group motors and detectors, respectively, in the global scope of IPython [*cf.* Figs. 1 ▸, 4 ▸ and 3 ▸(*a*)], just like how RE is recommended by *Bluesky* developers for the RunEngine instance in the same scope. We find this much more natural as a way to mark the ‘movability’ of devices than alternatives, like passing two *ad hoc* dictionaries as arguments to server_start which has the disadvantage that the device lists cannot be modified dynamically. We have even gone one step further and modified core *Ophyd* code to allow device object names like M.m1 that contain dots, so that we can enforce a policy that the name of a device object must be a Python expression referencing exactly the same object, which has proven to again save quite a lot of code.

## 3. Further plans on *Mamba*

After quite extensive research on the eligibility of *Bluesky* for the applications at HEPS, we concluded that, apart from the lack of GUI integration, *Bluesky* is able to cover most low-frequency low-throughput (typically with <10 Hz communication between the computer and devices involved, and data rates <100 MB s^−1^) needs at HEPS, in both regular ‘step scan’ experiments and certain preparation steps (*e.g.* the automated arming of samples, including the fine tuning of their positions; *cf.* also the requirement from the B5 beamline at HEPS mentioned in Section 2). Nevertheless, because HEPS is a fourth-generation synchrotron radiation facility, its small X-ray spots and high brightness not only facilitates high-resolution imaging but also necessitates continuous scans (fly scans) to handle the significantly larger number of data points and the much more serious radiation damage of samples. So both high-frequency and high-throughput applications – exactly where *Bluesky* is currently not very good – are essential to HEPS; if these weaknesses are somehow addressed, *Bluesky* will be able to provide a solid unified basis for beamline experiments at HEPS.

We begin with high-frequency applications, represented by fly scans; in comparison with fly scans, a typical high-frequency (but low throughput) application is sound recording. When performing fly scans (sound recording), because regular computers cannot handle the influx of data points at too high frequencies, we instead use dedicated controllers (sound recorders) to do the handling; the computer reads data from the controllers in a block-by-block (instead of point-by-point) fashion, and other than that just sends control messages like ‘start’, ‘stop’ or ‘pause’. From the above we can see that the key to high-frequency applications is asynchronous control (indirectly with dedicated controllers), which currently does not seem quite easy to do with *Bluesky*’s RunEngine. In fact the latter already has primitive support for simple fly scans, which only need ‘start’ (the kickoff operation in RunEngine) and ‘stop’ (the complete operation), where the data readout is mainly carried out offline (the collect operation run after complete). At HEPS, we are currently exploring an implementation of fly scans that allows real-time tuning of the scanning behaviors (adaptive speed/step-size tuning, automatic pausing/resuming *etc.*) based on online processing of the data read from controllers. This might be of particular interest in requirements like obtaining the optimal X-ray spot size and wavefront for focus alignment, as well as enabling ultra-high stability of the sample and X-ray probe during multi-dimensional scanning measurements at the hard X-ray nanoprobe beamline (B2) at HEPS.

**Figure 5:**
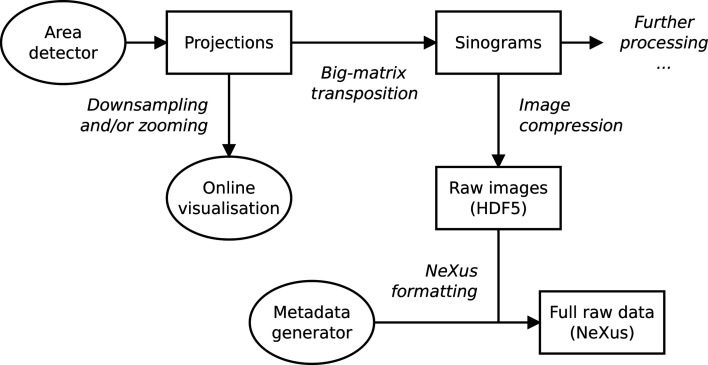
A partial data-pipe graph for a simple high-throughput tomography experiment.

*Bluesky* supports online data processing using Python packages from the *SciPy* ecosystem (Virtanen *et al.*, 2020 ▸), but this is currently carried out through synchronous point-by-point callbacks; similar to how point-by-point processing of sound data cannot be carried out synchronously with regular computers, synchronous processing is unsuitable for high-throughput applications. The solution is also similar – use asynchronous processing instead; noticing the discussion in the previous paragraph, we can see that the same asynchronous mechanism we envision also covers the data-processing needs for high-frequency experiments naturally. To address the complexity in both the asynchronous collaboration of worker processes (buffering, polling/pushing, error handling *etc.*, plus the resource management for processing of big data) and the diverse domain-specific logics we need to support, we are developing what we call the *Mamba Data Worker* (*MDW*) framework, which will be to an extent like *HiDRA* (Fischer *et al.*, 2017 ▸) and *Odin* (Yendell *et al.*, 2017 ▸). However, instead of focusing more on data producers, *MDW* will treat producers and consumers equally, also handling the diverse formatting requirements of raw data from beamline experiments at HEPS (Hu *et al.*, 2021*a* ▸). The same requirements are, to our knowledge, not something actively pursued by *Bluesky*’s *databroker*, aside from the problem we notice that *databroker* is not performant enough when there is heavy disk I/O on the same machine. Moreover, instead of mainly supporting linear processing pipelines, *MDW* will support full-fledged graphs of data pipes (Fig. 5 ▸), perhaps implemented in cooperation with the *Daisy* project (Hu *et al.*, 2021*b* ▸); this is crucial for the real-time tuning of fly scans and will also be very helpful in complex multimodal experiments.

As has also been discussed by Hu *et al.* (2021*a* ▸), aside from the diverse needs for processing of ‘real’ data from experiments, the challenges posed by the many types of experiments possible at HEPS also include the complexity in management of the scientific metadata for these experiments, which will directly affect the formatting of raw data by *MDW*. From the data producers’ side, for each type of experiment at a certain beamline, the number of parameters intended to be tuned by users (especially typical users) is usually much smaller than the total number of parameters for devices accessible on the beamline. Therefore it is also necessary to extend *Bluesky*’s RunEngine for each type of beamline experiment, so that users’ needs to care about irrelevant parameters are systematically minimized. Noticing the strong correlation between the specification of scientific metadata, device parameters and data-pipe graphs, we will be designing an experiment parameter generator (EPG) mechanism. Given an experiment schema, the EPG should accept a minimized group of inputs; while the inputs are selected for typical needs, the output should be in a form both customisable by advanced users and friendly to automation mechanisms. In the same spirit of simplifying user operations, we will also develop *Mamba* modules to automate experiment-specific preparation steps, as are mentioned in the beginning of this section; considering the small X-ray spots allowed at HEPS, this will help greatly in reducing the time costed by tasks like the fine tuning of beams. We find that ‘intelligent’ techniques, *e.g.* those based on statistical learning, are often useful in these steps, and we believe the architecture of *Mamba* will help to incorporate these techniques into our workflow.

For both the backend and frontend of *Mamba*, we fully realize the necessity to reuse off-the-shelf code from open-source projects to avoid unnecessary duplication of efforts in providing domain-specific abstractions. Integration of code from projects, *e.g.* *xrayutilities* (Kriegner *et al.*, 2013 ▸) and *diffcalc* (https://github.com/DiamondLightSource/diffcalc, for *SPEC*-like access to crystallographic coordinates) on the backend side, as well as *TomoPy* (Gürsoy *et al.*, 2014 ▸) and *pyFAI* (Ashiotis *et al.*, 2015 ▸) (for requirements in tomography) on the frontend side, is already under investigation. We also realize the dominant status of certain projects in specific fields, *e.g.* *MXCuBE* (Oscarsson *et al.*, 2019 ▸) in macromolecular crystallography, and will consider ways to provide a smooth experience at HEPS to users familiar with these projects. We may reuse *Mamba* components just enough to integrate the upstream project into the workflow at HEPS, or conversely integrate upstream components into *Mamba*, or even (if preferable) reimplement functionalities in *Mamba* and just emulate the GUIs for them; the actual way will be chosen depending on the specific project, in friendly cooperation with upstream developers, with the goal of minimizing the efforts on both sides in mind. We also note the necessity to support control systems other than the *Experimental Physics and Industrial Control System* (*EPICS*, https://epics-controls.org/), which is certainly doable with *Bluesky* but just not a focus to its developers. This is imperative for high-throughput area detectors, which are non-trivial to support cleanly with the *areaDetector* framework in *EPICS* (Rivers, 2010 ▸), and we are working on direct *Ophyd* support for them with *MDW* integration. There may also be systematic demand for other devices unsupported by *EPICS* that cannot be replaced with supported workalikes, which has fortunately not yet been encountered by us.

**Figure 6:**
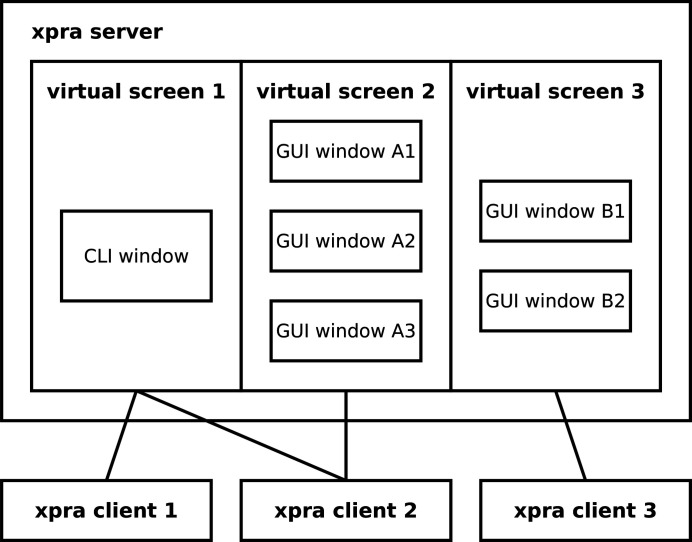
*xpra* provides virtual screens, each of which can be accessed by multiple clients simultaneously.

To reduce the efforts necessary for implementation and integration of GUIs, we are making what we call *Mamba GUI Studio* (*MGS*), to provide reusable utility widgets and to allow drag-and-drop composition of high-level GUI components, similar to what is carried out by Sobhani & Vescovo (2020 ▸). A feature commonly requested for *Mamba* is cross-platform use of its frontend, but we find it really complex to expose its RPC service (especially the command-injection mechanism) to the network without harming security. For this reason, we only allow the backend and frontend of *Mamba* to run on the same host, but meanwhile we plan to use the *xpra* utility (https://xpra.org/) (Fig. 6 ▸), with communication secured with SSH, to provide access on remote computers with operating systems supported by *xpra*. Since *xpra* supports multiple coexisting ‘virtual screens’ and simultaneous access to one virtual screen by multiple clients, users can additionally collaborate either using self-chosen groups of GUIs or using GUI groups shared by others; proper coordination is obviously needed between users, perhaps using walkie-talkies or some chat software, to avoid conflicts between their operations.

## 4. Conclusions

We are developing a *Bluesky*-based *Mamba* software framework for the diverse experiment requirements at HEPS. We use command injection with feedback to allow the command line interface and graphical user interfaces of *Mamba* to complement each other constructively; considering that certain functionalities are unsuitable for command injection, *Mamba* provides a remote-procedure-call service, which also supports proactive pushing of status updates. We take multiple measures to further simplify the codebase of *Mamba*, like the use of ZError to simplify error handling, and the use of M and D to group motors and detectors, respectively. We find *Bluesky*’s weaknesses in high-frequency and high-throughput experiments to be exactly where it lacks in requirements at HEPS, and plan to address the former by improving *Bluesky*’s support for asynchronous control. To fully address the complexity in data processing for high-throughput experiments, we are developing *Mamba Data Worker*, which will cover the entire process from producers to consumers, as well as support full-fledged graphs of data pipes. To simplify the specification of scientific metadata, device parameters and data-pipe graphs, we will develop an experiment parameter generator; the generation will be tailored systematically according to the experiment type, and its output will be customisable yet machine friendly. Experiment-specific modules to automate preparation steps will be developed similarly. We are investigating the integration of off-the-shelf code into *Mamba* to provide domain-specific functionalities, and are also developing *Mamba GUI Studio* to simplify the implementation and integration of graphical user interfaces. We plan to use an *xpra*-based mechanism to allow cross-platform access to *Mamba*, which will also enable easy collaboration between users.
